# Divergence of Dioecious *Hippophae tibetana* Endophytic Communities and Investigation of Their Key Driving Factors

**DOI:** 10.3390/microorganisms14061211

**Published:** 2026-05-27

**Authors:** Yifan Mao, Dawei Chen, Kun Sun

**Affiliations:** College of Life Sciences, Northwest Normal University, Lanzhou 730070, China; maoyifan202310@163.com (Y.M.); kunsun@nwnu.edu.cn (K.S.)

**Keywords:** dioecious plant, endophyte, divergence, driving factors, *Hippophae tibetana*

## Abstract

Dioecious plant species display sexual dimorphism in terms of their morphological and physiological properties. However, little is known about the differentiation among endophytes within female versus male members of dioecious host plants. Hence, the endophyte diversity and composition of different tissues of male and female *Hippophae tibetana* were investigated using amplicon sequencing, and key factors driving the differences were determined. The results showed that there were divergences in endophytic diversity, community composition, connectivity and complexity of the co-occurrence network between females and males *H. tibetana*. The females and males owned their unique phyla of endophytic bacteria (Fusobacteriota and Chloroflexi, respectively). Significant enrichment of species at different levels was found between females and males, suggesting that these species could be potential biomarkers for male and female *H. tibetana*. Variance partitioning analysis (VPA) and Spearman analysis revealed that the phytostoichiometry and metabolites of *H. tibetana* explained more differences in community composition of fungal and bacterial endophytes than rhizosphere soil physicochemical properties, and endophytes exhibited a significant positive correlation with the phytostoichiometry and metabolites of *H. tibetana*. PICRUSt and FUNGuild predictive analyses revealed differences in endophytic fungal function between female and male *H. tibetana*, while the endophytic bacterial functions were metabolism. These results reveal the sexual differentiation of endophytes in dioecious plants and provide important knowledge for dioecious plant–microbe interactions.

## 1. Introduction

Dioecious plant species are extensively distributed across diverse phyla, spanning from bryophytes and gymnosperms to angiosperms. They constitute a crucial component of terrestrial ecosystems [1], which have evolved independently from hermaphroditic ancestors, including 15,600 species in 987 genera and 175 families [2]. Previous studies have reported that male and female plants exhibit sexual dimorphism in growth, defense, and resource allocation [3]. Due to the relatively greater reproductive effort in comparison with males [4], female plants have been found to necessitate more favorable habitats for their survival [5]. In comparison with female plants, male plants exhibit a greater resistance or tolerance to diverse stressful environments [6]. Yu et al. [7] discovered that male-biased *Populus euphratica* populations demonstrate a superior adaptation compared with female plants under relatively dry and infertile soil conditions. Jiang et al. [8] have reported that male *P. cathayana* exhibit a greater tolerance to enhanced UV-B radiation compared with females. Recently, the differences between male and female plants have mainly focused on morphology, resource allocation, reproductive allocation, stress adaptive capacity, and nutrient availability in stressful environments, which have emerged as a topic of significant interest [9,10]. Although these research results have laid a certain theoretical and practical foundation for revealing the environmental adaptation mechanisms of dioecious plants, a unified understanding has not yet been formed. This is mainly because the research focus is often relatively narrow and lacks comprehensive and systematic studies on the influence of multiple factors on the adaptation of dioecious plants to the environment, especially the influence of the plant microbiome. This will help us reveal the environmental adaptation mechanisms of dioecious plants from a completely new perspective.

Throughout the course of plant evolution, the plant microbiota, also known as the microbiome, has coevolved with its host over millions of years. This coevolutionary relationship is intricately linked to plant fitness, with the plant and its associated microbiota functioning as a “holobiont” [11]. They form intricate interactions with plants and perform a variety of life-sustaining functions for their host. These functions include facilitating nutrient uptake, augmenting resistance to abiotic stresses, and preventing diseases [12]. These plant microbiota, also known as the microbiome, inhabit various niches, including the plant surface (e.g., phyllosphere microorganisms), within plant tissues (endophytes), and are acquired from the environment (e.g., rhizosphere microorganisms) [13]. Among these, endophytes serve as the core of the plant-associated microbiome. They exhibit a closer association with plant growth, promoting the accumulation of host secondary metabolites and nutrient transformation. Notably, they play a crucial role in plant stress tolerance [13]. Meanwhile, Guo et al. [14] reported that sex exerted distinct impacts on the microbial assembly within each niche. In particular, fungal endophytes exhibited significant disparities in community structure, keystone species, and community complexity between male and female *P. cathayana*. However, little is known about the differentiation of endophytes in dioecious plants and their key driving factors. Consequently, exploring the diversity and community structure of endophytes in dioecious plants is crucial for uncovering the differences in environmental adaptability among dioecious plants.

*Hippophae tibetana* is a perennial shrub with dioecious and parthenogenetic clonal reproduction and is a pioneer species in community succession with important ecological value. Meanwhile, as a medicinal and edible homologous plant in China, *H. tibetana* contains a diverse array of bioactive compounds in its fruits, including flavonoids, polyphenols, vitamins, carotenoids, polysaccharides and other biologically active substances [15]. These bioactive constituents contribute to a range of pharmacological activities, such as anti-inflammatory, antioxidant, antitumor, and hepatoprotective effects [16]. It is distributed across the Tibetan Plateau and Himalayan habitat at altitudes of 2800–5200 m [17]. Previous studies have shown that male and female *H. tibetana* have different morphologies, physiologies and adaptive strategies in this dioecious species [18]. However, as dioecious plants, the different adaptation strategies of female and male plants to environments in terms of endophytes have been little studied. The aim of this study was to explore whether there are differences in endophytes between females and males of the dioecious *H. tibetana* and to determine if there are key factors driving the differences. Thus, in this study, the endophytes within different tissues of male and female *H. tibetana* were investigated using amplicon sequencing, and key factors driving the differences were determined. Our findings may lay a foundation for comprehending plant–microbiome interactions and offer crucial insights into the relationship between the environmental adaptation differences of dioecious plants and microorganisms.

## 2. Materials and Methods

### 2.1. Site Location and Sampling

In September 2023, the soil samples were collected from two habitats at Tianzhu County, Wuwei City, Gansu Province, China (102°45′11.26″ E, 37°12′47.50″ N, 2903.56 m). The average annual precipitation is 410.50 mm. The average annual potential evaporation is 1592 mm, which is 3.8 times the annual precipitation. The average annual temperature is −0.1 °C. The total number of annual sunshine hours is 2600 h. In order to explore whether there are differences in the endophytes and their key driving factors, we selected two habitats, which, in all cases, were at a distance of 5 km apart. Habitat A was located near a beach (Figure S1a), and habitat B was located in a meadow (Figure S1b); the rhizosphere soil physicochemical properties of the two habitats are shown in Table 1. The samples were randomly collected for each category (roots, stems and leaves between females and males of the dioecious *H. tibetana* in different habitats). The leaves, stems, and roots (coarse roots) of *H. tibetana* (basal diameter = 0.47 ± 0.08 cm, plant height = 23.42 ± 1.32 cm, and crown width = 184 ± 4.56 cm^2^) were meticulously collected in accordance with uniformity criteria based on size. Each individual sample was composed of 0.5 g of each plant part, with three biological replicates collected for each group (sample code: AMR represents the male endophyte of the dioecious *H. tibetana* root in habitat A; AFR represents the female endophyte of the dioecious *H. tibetana* root in habitat A; AMS represents the male endophyte of the dioecious *H. tibetana* stem in habitat A; AFS represents the female endophyte of the dioecious *H. tibetana* stem in habitat A; AML represents the male endophyte of the dioecious *H. tibetana* leaf in habitat A; AFL represents the female endophyte of the dioecious *H. tibetana* leaf in habitat A; BMR represents the male endophyte of the dioecious *H. tibetana* root in habitat B; BFR represents the female endophyte of the dioecious *H. tibetana* root in habitat B; BMS represents the male endophyte of the dioecious *H. tibetana* stem in habitat B; BFS represents the female endophyte of the dioecious *H. tibetana* stem in habitat B; BML represents the male endophyte of the dioecious *H. tibetana* leaf in habitat B; and BFL represents the female endophyte of the dioecious *H. tibetana* leaf in habitat B). The samples were promptly placed in an ice box and transported to the laboratory without delay (within 3 h). The samples were carefully separated and thoroughly washed with running tap water, followed by three rinses with distilled water. To ensure the surface sterilization of the plant parts, the root samples were sequentially immersed in 70% ethanol for 5 min, 2.5% sodium hypochlorite for 1–2 min, and 70% ethanol for 1 min, and subsequently rinsed five times with sterile Millipore water. To validate the sterilization efficiency, the final portion of the washing water was inoculated in potato dextrose agar (PDA), incubated at 28 °C for 10 d, as well as in nutrient agar (NA), incubated at 37 °C for 3 d. All samples were stored at −80 °C until DNA extraction.

### 2.2. Rhizosphere Soil Sampling and Measurement of Soil Physicochemical Properties

Plant roots were carefully removed from the soil, and most of the soil was shaken off, leaving a thin soil layer of approximately 1 mm attached to the roots. A sterilized brush was then used to collect the soil attached to the roots [19]. Only soil samples were used to determine physicochemical properties.

Soil pH value was measured using the potentiometric method [20]; soil organic matter content using the high-temperature dry combustion method [21]; available nitrogen by the diffusion absorption method [22]; available phosphorus by the molybdenum–antimony colorimetric method [23]; available potassium by ammonium acetate extraction with flame photometry [24]; total nitrogen by the Kjeldahl method [25]; total phosphorus by the digestion–molybdenum–antimony anti-spectrophotometry method [26]; total potassium by the hydrofluoric acid digestion method [27]; the salt content of the soil by the conductivity method [28]; and the water content of the soil by the gravimetric method [29].

### 2.3. DNA Extraction, Polymerase Chain Reaction (PCR) Amplification, and Sequence Processing

For rinsing and surface sanitization, 0.5 g of each tissue sample was collected. Three replicates of a single sample were utilized to extract endophyte DNA from each tissue of the plant. Genomic DNA from all samples was extracted using the MOBIO Power-Soil^®^ Kit (MOBIO Laboratories, Inc., Carlsbad, CA, USA), strictly following the manufacturer’s instructions. The concentration of the DNA extracts was measured using a NanoDrop spectrophotometer (Thermo Fisher Scientific, Model 2000, Waltham, MA, USA). Subsequently, the extracts were stored at −20 °C for subsequent PCR amplification. For the amplification of the bacterial 16S gene (amplification length: 470 bp), the primers 341F (5′-CCTACGGGNGGCWGCAG-3′) and 805R (5′-GACTACHVGGGTATCTAATCC-3′) were employed. PCR was performed under the following conditions: an initial denaturation step was conducted at 95 °C for 3 min. Subsequently, 30 cycles were carried out. Each cycle comprised three steps: denaturation at 95 °C for 30 s, annealing at 52 °C for 30 s, and extension at 72 °C for 45 s. Finally, a final extension was executed at 72 °C for 5 min [30]. Regarding the amplification of fungal ITS genes (amplification length: 350 bp), the primers ITS1F (5′-CTTGGTCATTTAGAGGAAGTAA-3′) and ITS2R (5′-GCTGCGTTCTTCATCGATGC-3′) were utilized. PCR was performed using the following thermal cycling conditions: initial denaturation at 95 °C for 3 min; followed by 35 amplification cycles, each consisting of denaturation at 95 °C for 30 s, annealing at 55 °C for 30 s, and extension at 72 °C for 45 s; and a final extension step at 72 °C for 10 min [30]. The PCR products were analyzed via agarose gel electrophoresis. The PCR products from all samples were pooled and purified using the EasyPureTM PCR Cleanup/Gel Extraction Kit (Axygen Biosciences, Union City, CA, USA), according to the manufacturer’s instructions. Subsequently, the purified PCR products were sequenced (Paired_End) on an Illumina NovaSeq 6000 platform (Illumina, Inc., San Diego, CA, USA).

### 2.4. Measurement of Plant Phytostoichiometry and Metabolites

The total flavonoid content of all samples was analyzed using the AlCl_3_ colorimetric method [31], the total polysaccharides content was determined by the phenol–sulfuric acid method [32], and the total polyphenols content of all samples was analyzed using the Folin–Ciocalteu colorimetric method [33]. Total nitrogen was determined by the Kjeldahl method [34], total phosphorus was determined by the digestion–molybdenum–antimony anti-spectrophotometry method [26], and total carbon was determined by the potassium dichromate–sulfuric acid oxidation method [29].

### 2.5. Data Analysis

The fungal internal transcribed spacer (ITS) sequences, bacterial 16S ribosomal RNA (16S rRNA) genes, and fungi were subjected to analysis using the QIIME 2 (v 2024.10) software [30]. Fungal and bacterial sequences were trimmed and allocated to each sample according to their barcodes. The UPARSE-OUTref method was employed to classify operational taxonomic units (OTUs) at the species level by conducting a search of all sequences against the Silva bacterial 16S database (https://www.arb-silva.de/ (accessed on 4 January 2026), v138.2) [21,24]. OTUs were classified at the species level through a search against the UNITE fungal database (https://unite.ut.ee/ (accessed on 4 January 2026), v10.0) [35]. Sequences were grouped into OTUs at a 97% similarity level by utilizing the USEARCH software v12 (http://drive5.com/uparse/ (accessed on 6 January 2026) [36]. Rarefaction analysis, implemented using Mothur (version v1.43.0), was performed to unveil the diversity indices, encompassing Good’s coverage, Chao 1, and Shannon [37]. Significant differences in the endophytic communities between sex and habitat were analyzed using permutational multivariate ANOVA (PERMANOVA) using the adonis function of the vegan package. The principal coordinates analysis (PCoA) was performed to visualize differences in the endophytic community composition using the PCoA function of the ade4 package in R v4.5.1 [38]. Linear discriminant analysis effect size (LEfSe) biomarker analysis was employed to investigate the disparities in species composition and functional composition among the samples [39]. Linear discriminant analysis effect size (LDA effect size (LEfSe)) (http://huttenhower.sph.harvard.edu/galaxy (accessed on 16 January 2026) was employed to identify microbial community biomarkers (Wilcoxon test, *p* < 0.05; log LDA score > 2.0) [40]. A microbial co-occurrence network analysis among the genera with a relative abundance of >0.1% was performed with the Pearson correlation (a correlation threshold of 0.6 and a significance level of 0.01) and Euclidean distance using the CoNet plugin in Cytoscape (v3.3.0). gephi (v0.9.2) was used to visualize the network, and topological parameter calculations were performed by Network Analyzer [41]. Variance partitioning analysis (VPA) was utilized to calculate the contributions of plant metabolites, the physical and chemical properties of the rhizosphere soil, and their interactions to the variation in the fungal and bacterial endophytic communities with the vegan package [42]. The Spearman method was adopted for the correlation analysis between metabolites and endophytes [43]. Ecological functions were annotated using PICRUSt v2.6.2 for the 16S rDNA OTU and FUNGuild v1.0 for the ITS OUT [44]. The data were analyzed by the SPSS16.0 software for variance (one-way ANOVA) and Duncan’s multiple-range test (*p* < 0.05).

## 3. Results

### 3.1. Surface Sterilization Efficiency and Rarefaction Curve

The results showed that no colonies were observed in the PDA and NA medium after a certain period of cultivation, which reflected that the method of surface sterilization was effective, and the surface-sterilized samples could be used for subsequent tests.

The rarefaction curve can reflect the variation in species diversity and the richness of samples with the sequencing amount. With the increase in the amount of sequencing effort, the rarefaction curves of the samples based on the number of species observed became stable. When the sequencing depth reached 20,000, all curves tended to flatten, which indicated that the amount of sequencing data was gradually becoming reasonable (Figure S2).

### 3.2. Analysis of Alpha Diversity

**Figure 1:**
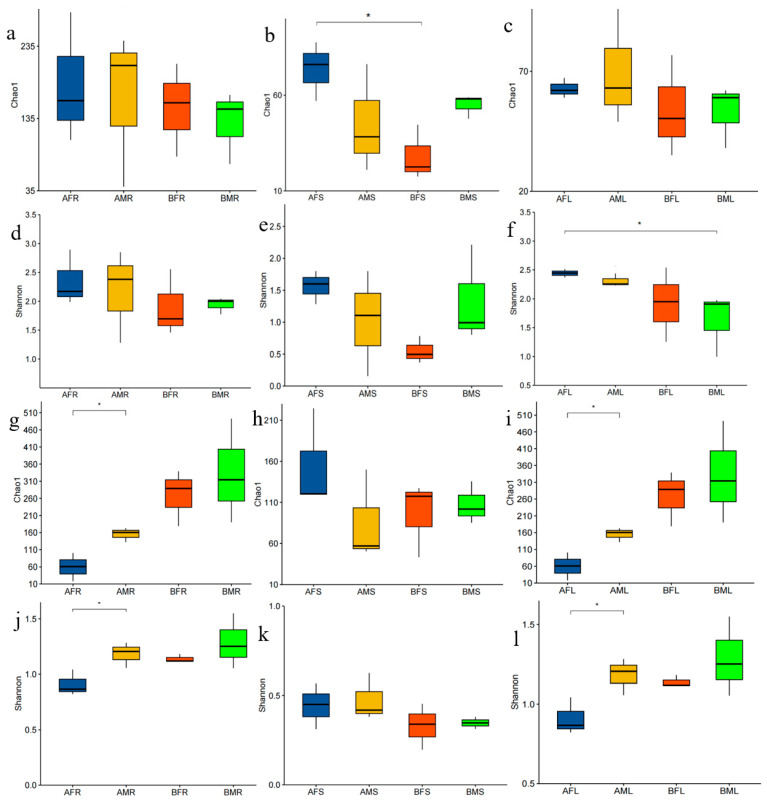
Alpha diversity of endophyte between males and females of dioecious *H. tibetana* in different habitats. Note: (**a**) represents Chao1 index of root endophytic fungi; (**b**) represents Chao1 index of stem endophytic fungi; (**c**) represents Chao1 index of endophytic fungi in leaves; (**d**) represents Shannon index of root endophytic fungi; (**e**) represents Shannon index of stem endophytic fungi; (**f**) represents Shannon index of endophytic fungi; (**g**) represents Chao1 index of root endophytic bacteria; (**h**) represents Chao1 index of stem endophytic bacteria; (**i**) represents Chao index of endophytic bacteria in leaf; (**j**) represents Chao1 index of root endophytic bacteria; (**k**) represents Shannon index of stem endophytic bacteria; (**l**) represents Shannon index of endophytic bacteria. “*” represents a significant difference at the 0.05 level.

In the females and males of the dioecious *H. tibetana* root, the endophytic fungal and bacterial Chao1 and Shannon indices of the AFR sample were lower than those of the AMR sample, respectively (Figure 1a,d,g,j). The endophytic fungal Chao1 index of the BFR sample was higher than that of the BMR sample (Figure 1a), while the endophytic bacterial Chao1 index was the opposite (Figure 1g). The endophytic fungal and bacterial Shannon indices of the BFR sample were lower than those of the BMR sample, respectively (Figure 1d,j).

In the females and males of the dioecious *H. tibetana* stem, the endophytic fungal and bacterial Chao1 and Shannon indices of the AFS sample were higher than those of the AMS sample, respectively (Figure 1b,e,h,k). The endophytic fungal Chao1 index of the BFS sample was lower than that of the BMS sample (Figure 1b), while the endophytic bacterial Chao1 index was the opposite (Figure 1h). The endophytic fungal and bacterial Shannon indices of the BFS sample were lower than those of the BMS sample, respectively (Figure 1e,k).

In the females and males of the dioecious *H. tibetana* leaf, the endophytic fungal and bacterial Chao1 indices of the AFL sample were lower than those of the AML sample, respectively (Figure 1c,i). The endophytic fungal Shannon index of the AFL sample was higher than that of the AML sample (Figure 1f), while the endophytic bacterial Shannon index was the opposite (Figure 1l). The endophytic fungal and bacterial Chao1 indices of the BFL sample were lower than those of the BML sample (Figure 1c,i). The endophytic fungal Shannon index of the BFL sample was higher than that of the BML sample (Figure 1f), while the endophytic bacterial Shannon index was the opposite (Figure 1l).

**Figure 2:**
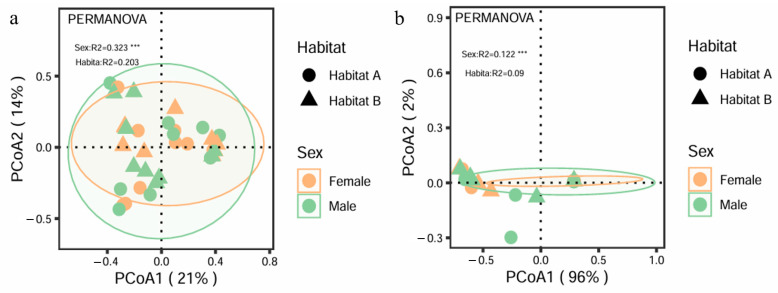
The principal coordinates analysis (PCoA) plots of the variation in endophyte fungal (**a**) and bacterial (**b**) communities by different factors. Note: “***” represents a significant difference at the 0.001 level.

The PCoA analysis separated samples by different sex and habitat; the PERMANOVA test indicated a significant difference in endophyte fungal and bacterial community composition between sex (male and female) and habitat (habitat A and habitat B) (Figure 2). We found that sex had more influence on fungal (R^2^ = 0.323; *p* = 0.001) and bacterial (R^2^ = 0.122; *p* = 0.001) community composition compared with habitat (Figure 2a,b).

### 3.3. Community Composition

**Figure 3:**
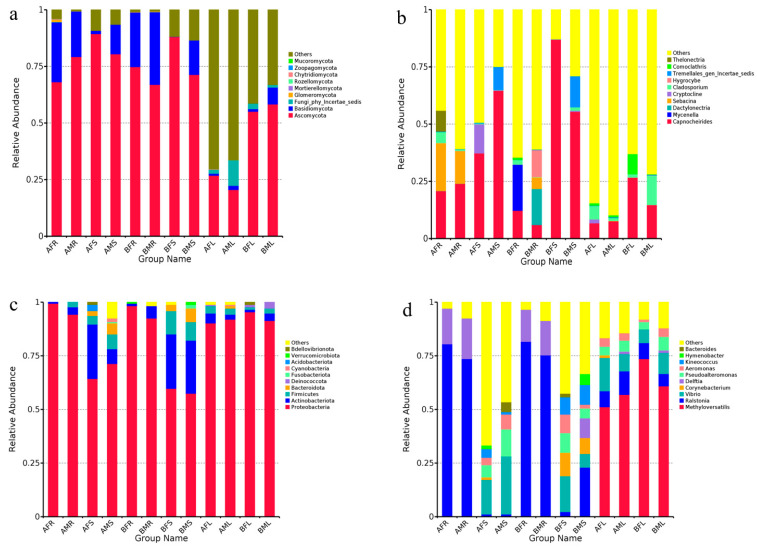
Relative abundances of the endophyte between males and females of dioecious *H. tibetana* in different habitats. Note: Endophytic fungi at the phylum level (**a**), endophytic fungi at the genus level (**b**), endophytic bacteria at the phylum level (**c**), and endophytic bacteria at the genus level (**d**). “Other” represents the total relative abundance outside the top ten maximum relative abundance levels.

The fungal OTUs were assigned to 16 phyla and 576 genera. The dominant fungal phylum across all samples was Ascomycota, with relative abundances ranging from 20.55% to 89.39% (Figure 3a). At the genus level, *Capnocheirides* was the dominant fungal genus in the AFR, AMR, AFS, AMS, BFS, BMS, AFL, AML, BFL and BML samples, with relative abundances of 6.02%~86.97%. *Mycenella* was the dominant fungal genus in the BFR samples (20.08%), and *Dactylonectria* was the dominant fungal genus in the BMR samples (15.74%) (Figure 3b).

The bacterial OTUs were assigned to 38 phyla and 608 genera. The dominant bacterial phylum across all samples was Proteobacteria, with relative abundances ranging from 57.47% to 99.42% (Figure 3c). At the genus level, *Ralstonia* was the dominant bacterial genus in the AFR, AMR, BFR, BMR and BMS samples, with relative abundances of 80.46%, 73.36%, 81.61%, 75.29% and 22.99%, while *Vibrio* was the dominant bacterial genus in the AFS, AMS and BFS samples, with relative abundances of 16.09%, 27.01% and 16.67%. *Methyloversatilis* was the dominant bacterial genus in the AFL, AML, BFL and BML samples, with relative abundances of 51.15%, 56.90%, 73.56% and 60.92% (Figure 3d).

**Figure 4:**
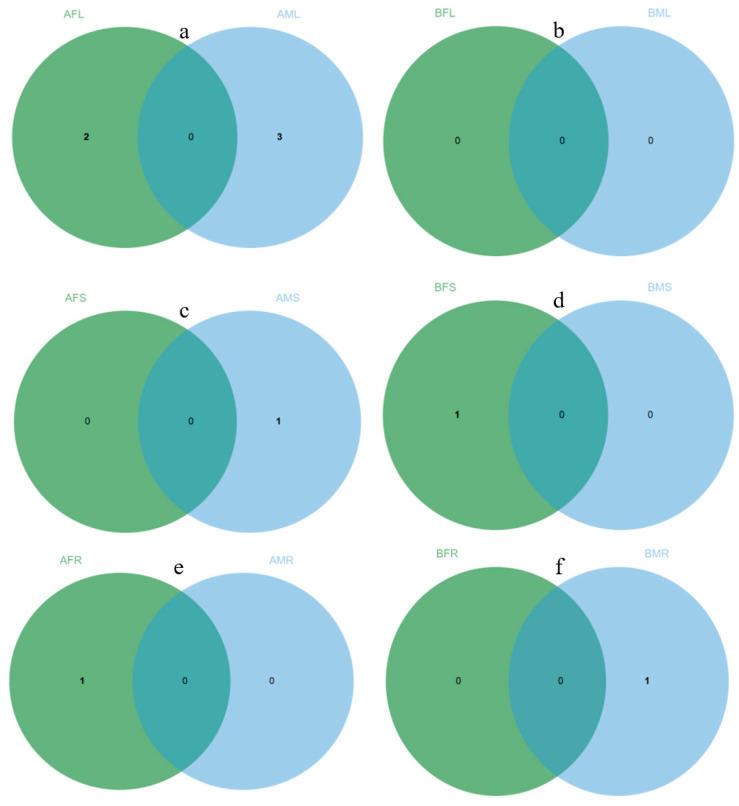
The shared and unique endophytic fungal phyla between males and females of dioecious *H. tibetana* in different habitats. Note: Endophytic fungal phyla between females and males of dioecious *H. tibetana* leaf in different habitat A (**a**); endophytic fungal phyla between males and females of dioecious *H. tibetana* leaf in different habitat B (**b**); endophytic fungal phyla between males and females of dioecious *H. tibetana* stem in different habitat A (**c**); endophytic fungal phyla between males and females of dioecious *H. tibetana* stem in different habitat B (**d**); endophytic fungal phyla between males and females of dioecious *H. tibetana* root in different habitat A (**e**); and endophytic fungal phyla between males and females of dioecious *H. tibetana* root in different habitat B (**f**).

With respect to the fungal phylum levels, two fungal phyla (Glomeromycota and Mortierellomycota) were unique in the AFL samples, and three fungal phyla (Rozellomycota, Chytridiomycota and Mucoromycota) were unique in the AML samples (Figure 4a). One fungal phylum (Mortierellomycota) was unique in the AMS samples (Figure 4c). One fungal phylum (Chytridiomycota) was unique in the BFS samples (Figure 4d). One fungal phylum (Rozellomycota) was unique in the AFR samples (Figure 4e). One fungal phylum (Chytridiomycota) was unique in the BMR samples (Figure 4f). Zero fungal phyla were shared in the AFL and AML, BFL and BML, AFS and AMS, BFS and BMS, AFR and AMR, and BFR and BMR samples (Figure 4). Zero fungal phyla were unique in the AFS, BFR, BMS, AMR, BFL and BML samples (Figure 4).

**Figure 5:**
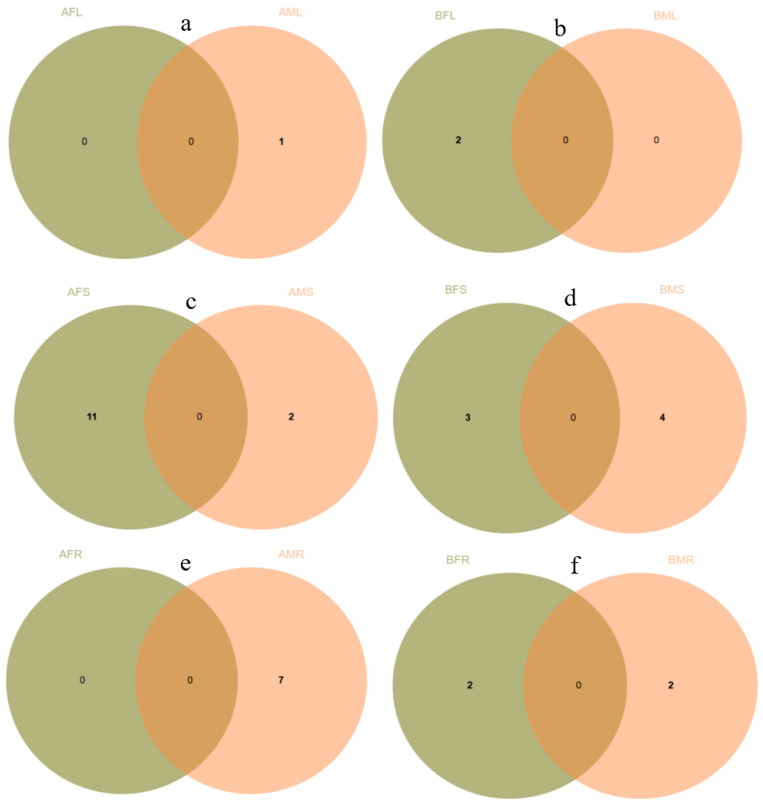
The shared and unique endophytic bacterial phyla between males and females of dioecious *H. tibetana* in different habitats. Note: Endophytic fungal phyla between bacteria and males of dioecious *H. tibetana* leaf in different habitat A (**a**); endophytic bacterial phyla between males and females of dioecious *H. tibetana* leaf in different habitat B (**b**); endophytic bacterial phyla between males and females of dioecious *H. tibetana* stem in different habitat A (**c**); endophytic bacteria phylum between males and females of dioecious *H. tibetana* stem in different habitat B (**d**); endophytic bacterial phyla between males and females of dioecious *H. tibetana* root in different habitat A (**e**); and endophytic bacterial phyla between males and females of dioecious *H. tibetana* root in different habitat B (**f**).

With respect to the bacterial phylum levels, one bacterial phylum (Bacteroidota) was unique in the AML samples (Figure 5a). Two bacterial phyla (Bacteroidota and Bdellovibrionota) were unique in the BFL samples (Figure 5b). Eleven bacterial phyla (Acidobacteriota, Chloroflexi, Latescibacterota, Verrucomicrobiota, Abditibacteriota, Entotheonellaeota, Bdellovibrionota, Aenigmarchaeota, Nitrospirota, Patescibacteria and Gemmatimonadota) were unique in the AFS samples, while two bacterial phyla (Desulfobacterota and Fusobacteriota) were unique in the AMS samples (Figure 5c). Three bacterial phyla (Myxococcota, Bdellovibrionota and Chloroflexi) were unique in the BFS samples, while four bacterial phyla (Acidobacteriota, Fusobacteriota, Verrucomicrobiota and Desulfobacterota) were unique in the BMS samples (Figure 5d). Seven bacterial phyla (Verrucomicrobiota, Acidobacteriota, Myxococcota, Patescibacteria, Planctomycetota, Desulfobacterota, Armatimonadota and Dependentiae) were unique in the AMR samples (Figure 5e). Two bacterial phyla (Fibrobacterota and Elusimicrobiota) were unique in the AFR samples, while two bacterial phyla (Deinococcota and Methylomirabilota) were unique in the BMR samples (Figure 5f). Zero bacterial phyla were shared in the AFL and AML, BFL and BML, BFS and BMS, AFS and AMS, AFR and AMR, and AFR and AMR samples (Figure 5). Zero bacterial phyla were unique in the AFL, BML and AFR samples (Figure 5a,b,e).

In brief, Chloroflexi and Fusobacteriota were unique endophytic bacteria phylum between male and female of dioecious *H. tibetana*, respectively (Table 2).

### 3.4. LEfSe Analysis

**Figure 6:**
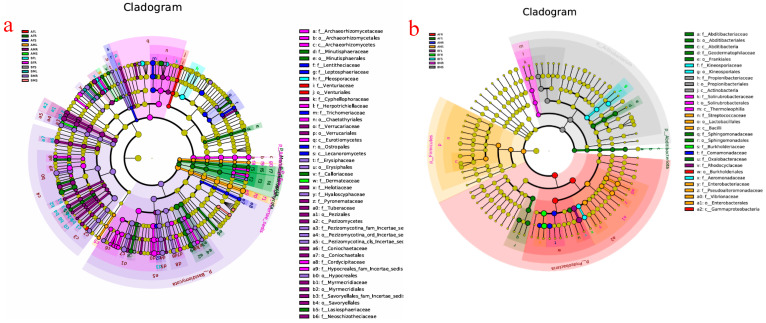
The linear discriminant analysis effect size (LEfSe) analysis between females and males of dioecious *H. tibetana* in different habitats. Note: Only taxa with LDA > 2 and Wilcoxon, *p* < 0.05. Endophytic fungi (**a**) and endophytic bacteria (**b**).

The LEfSe analysis showed differences in the root, stem and leaf bacterial and fungal communities between females and males of dioecious *H. tibetana* in different habitats. In the fungal community, there were significant differences at the family (Tuberaceae, Pyronemataceae, and Coniochatetales), order (Pezizales, Auriculariales, and Conniochaetales), and class (Pezizomycetes) levels in the AMR samples. There were significant differences at the phylum (Fungi_phy_Incertae_sedis), family (Fungi_fam_Incertae_sedis, Taphrinaceae, and Trichomeriaceae), and class (Rozellomycota_fam_ Incertae_sedis) levels in the AML samples. There were significant differences at the family (Plyeosporaceae and Leptosphaeriaceae) and class (Lecanoromycetes) levels in the AFS samples. There were significant differences at the family (Hyaloscyphhaceae) level in the BFL samples. There were significant differences at the phylum (Basidiomycota), family (Helotiales_fam_Incertae_sedis, Pezizomycotina_fam_Incertae_sedis, Hypocreales_fam_Incertae_sedis, Hygrophoraceae, Ceratobasidiaceae, Sebacinaceae and Hymenochaetales_fam_Incertae_sedis), order (Pezizomycotina_ord_ Incertae_sedis, Hypocreales, Xylariales, Cantharellales, Debacinales, and Hymenochaetales), and class (Pezizomycotina_cls_Incertae_sedis, Sordariomycetes and Agaricomycetes) levels in the BMR samples. There were significant differences at the family (Herpotrichiellaceae, Hypocreales_fam_Incertae_sedis, and Trichaeorhizomycetales) and order (Archaeorhizomycetales, Sordariales, Agaricales, Archaeorhizomyceyes and Eurotiomycetes) levels in the BFR samples. There were significant differences at the phylum (Glomeromycota), family (Sebacina_sp, Thelonectriaceae, Inocybaceae, and Glomeraceae), and order (Sebacinales, Telephorales, and Glomerales) levels in the AFR samples. There were significant differences at the family (Dermateaceae) level in the AMS samples. There were significant differences at the family (Venturiaceae) and order (Venturiales) levels in the AFL samples. There were significant differences at the family (Lycoperdaceae) level in the BFS samples. There were significant differences at the genus (*Genolevuria*) level in the BMS samples. There were significant differences at the genus (*Calycellina*) level in the BML samples (Figure 6a).

In the bacterial community, there were significant differences at the phylum (Proteobacteria), class (Gammaproteobacteria) and order (Burkholderiales) levels in the AFR samples. There were significant differences at the levels of genus (*Delftia* and *Glycomyces*), family (Comamonadaceae and Glycomycetaceae), and order (Glycomycetales) in the AMR samples. There were significant differences at the family (Chitinophagaceae and Solirubrobacteraceae), class (Thermoleophilia) and order (Solirubrobacterales and Chitinophagales) levels in the BMR samples. There were significant differences at the family (Rhodocyclaceae) and genus (Methyloversatilis) levels in the BFL samples. There were significant differences at the phylum (Firmicutes), family (Vibrionaceae, Enterobacteriaceae, and Aeromonadaceae), and order (Enterobacterales and Lactobacillales) levels in the AMS samples. There were significant differences at the family (Microbacteriaceae, Propionibacteriaceae, and Hymenobacteraceae) and order (Propionibacteriales and Cytophagales) levels in the BMS samples. There were significant differences at the phylum (Abditibacteriota), family (Sphingomonadaceae, Geodermatophilaceae, and Abditibacteriaceae), order (Sphingomonadales, Frankiales, and Abditibacteriales), and class (Abditibacteria) levels in the AFS samples. There were significant differences at the phylum (Actinobacteriota), class (Actinobacteria), order (Kineosporiales), and family (Kineosporiaceae and Streptococcaceae) levels in the BFS samples. There were significant differences at the family (Burkholderiaceae) and genus (*Ralstonia*) levels in the BFR samples (Figure 6b).

### 3.5. Co-Occurrence Network Analysis

**Figure 7:**
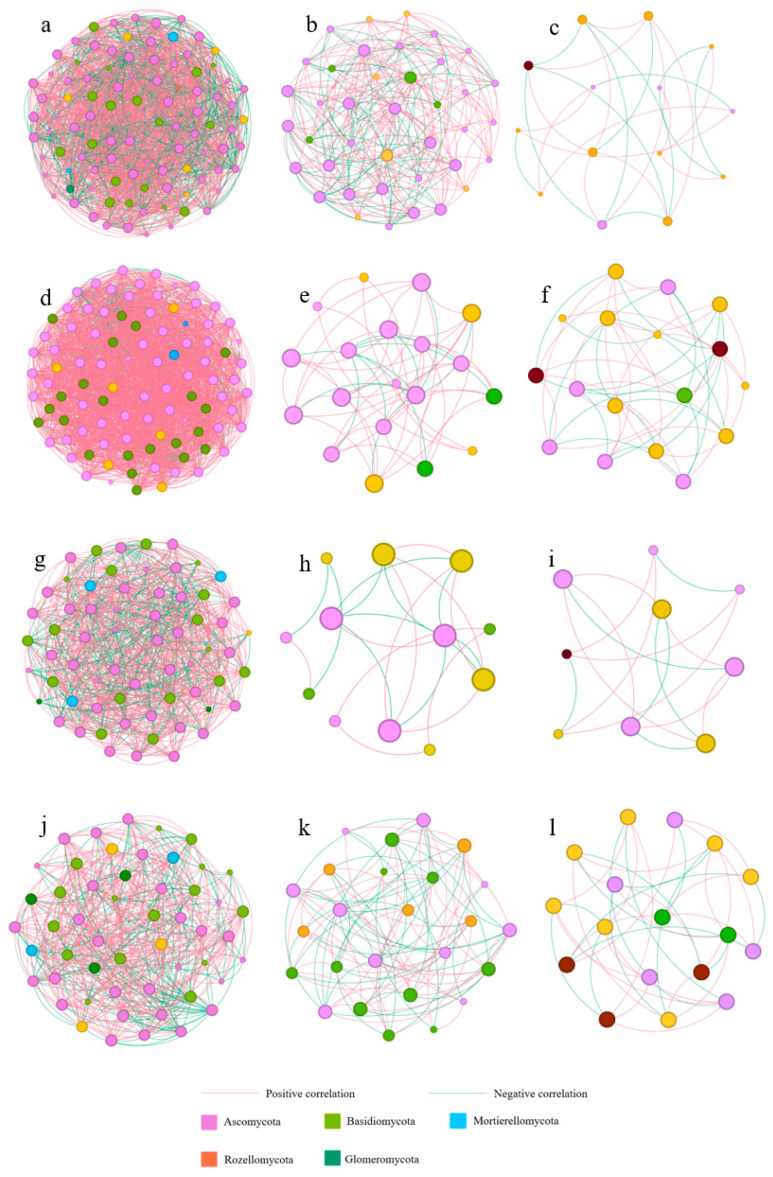
Co-occurrence network analysis of endophytic fungi between females and males of dioecious *H. tibetana* in different habitats. Note: Co-occurrence network diagram of endophytic fungi of female root in habitat A (**a**); co-occurrence network diagram of endophytic fungi of female stem in habitat A (**b**); co-occurrence network diagram of endophytic fungi of female leaf in habitat A (**c**); co-occurrence network diagram of endophytic fungi of male root in habitat A (**d**); co-occurrence network diagram of endophytic fungi of male stem in habitat A (**e**); co-occurrence network diagram of endophytic fungi of male leaf in habitat A (**f**); co-occurrence network diagram of endophytic fungi of female root in habitat B (**g**); co-occurrence network diagram of endophytic fungi of female stem in habitat B (**h**); co-occurrence network diagram of endophytic fungi of female leaf in habitat B (**i**); co-occurrence network diagram of endophytic fungi of male root in habitat B (**j**); co-occurrence network diagram of endophytic fungi of male stem in habitat B (**k**); and co-occurrence network diagram of endophytic fungi of male leaf in habitat B (**l**).

**Figure 8:**
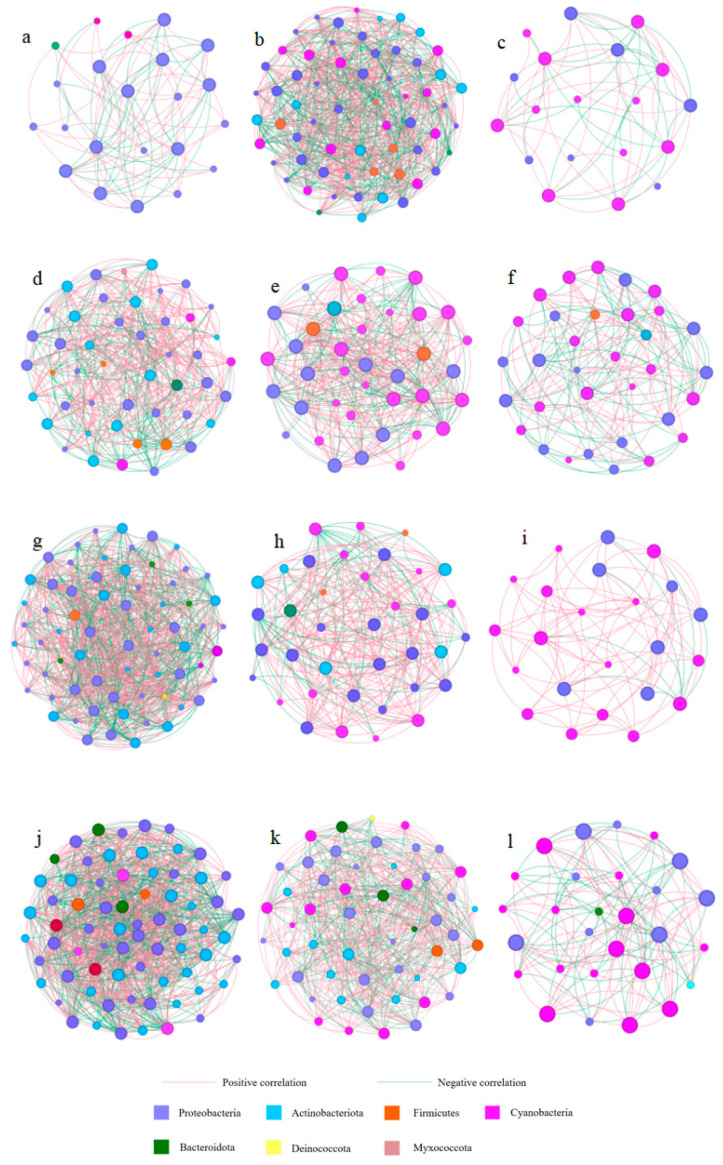
Co-occurrence network analysis of endophytic bacteria between males and females of dioecious *H. tibetana* in different habitats. Note: Co-occurrence network diagram of endophytic bacteria of female root in habitat A (**a**); co-occurrence network diagram of endophytic bacteria of female stem in habitat A (**b**); co-occurrence network diagram of endophytic bacteria of female leaf in habitat A (**c**); co-occurrence network diagram of endophytic bacteria of male root in habitat A (**d**); co-occurrence network diagram of endophytic bacteria of male stem in habitat A (**e**); co-occurrence network diagram of endophytic bacteria of male leaf in habitat A (**f**); co-occurrence network diagram of endophytic bacteria of female root in habitat B (**g**); co-occurrence network diagram of endophytic bacteria of female stem in habitat B (**h**); co-occurrence network diagram of endophytic bacteria of female leaf in habitat B (**i**); co-occurrence network diagram of endophytic bacteria of male root in habitat B (**j**); co-occurrence network diagram of endophytic bacteria of male stem in habitat B (**k**); and co-occurrence network diagram of endophytic bacteria of male leaf in habitat B (**l**).

As shown in Figure 7 and Figure 8, endophytic fungal and bacterial co-occurrence network connectivity and complexity showed differences between females and males of dioecious *H. tibetana* in different habitats (Table S1).

In the fungal community, the total number of nodes, links, positive edges, negative edges, relative modularity and map density of the AFR, AFL, BFS and BFL samples were lower than those of the AMR, AML, BMS and BML samples, respectively (Figure 7a,c,d,f,h,i,k,l; Table S1), while the total number of nodes, links, positive edges, negative edges, relative modularity and map density of the AFS and BFR samples were high than those of the AMS, BMS and BMR samples, respectively (Figure 7b,e,g,j; Table S1).

In the bacterial community, the total number of nodes, links, positive edges, negative edges, and relative modularity of the AFR, AFL, BFR, BFS and BFL samples were lower than those of the AMR, AML, BMR, BMS and BML samples, respectively (Figure 8a,c,d,f–l; Table S1), while the total number of nodes, links, positive edges, negative edges, and relative modularity of the AFS samples were lower than those of the AMS samples (Figure 8b,e; Table S1).

### 3.6. VPA and Spearman Correlation Analysis

**Figure 9:**
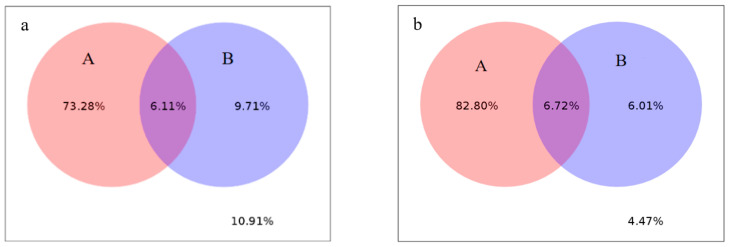
Variation partitioning analysis (VPA) of phytostoichiometry and metabolites versus rhizosphere soil physicochemical properties for endophytic fungal (**a**) and bacterial (**b**) community composition. Note: A: Phytostoichiometry and metabolites; B: physical and chemical characteristics of rhizosphere soil.

As shown in Figure 9, we discovered that phytostoichiometry and metabolites, and the rhizosphere soil physicochemical properties accounted for unique variations in the fungal (73.28% and 9.71%) and bacterial (82.80% and 6.01%) endophytic community composition of *H. tibetana*, respectively. The overlap of the two sets of predictors explained 6.11% and 6.72% of the variations in fungi and bacteria, respectively.

**Figure 10:**
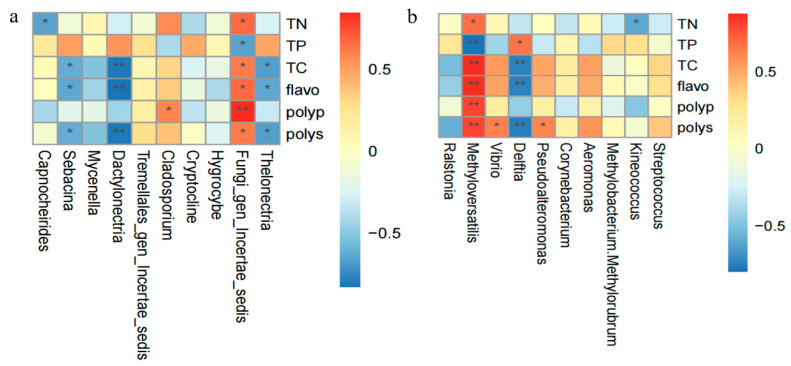
Correlation analysis between top ten dominant genera of endophytes and phytostoichiometry and metabolites. Note: (**a**) represents endophytic fungi. (**b**) represents endophytic bacteria. “*” represents that the differences are significant at *p* < 0.05; “**” represents that the differences are significant at *p* < 0.01. “−0.5 to 0.5” represents the R^2^ range. TN represents total nitrogen. TP represents total phosphorus. TC represents total carbon. Flavo represents total flavonoids. Polyp represents polyphenols. Polys represents polysaccharides.

The Spearman correlation analysis showed that Fungi_gen_Incertae_sedis was significantly positively correlated with total nitrogen, total carbon (*p* < 0.05), flavone (*p* < 0.05), polyphenol (*p* < 0.01) and polysaccharide (*p* < 0.05). *Cladosporium* was significantly positively correlated with polyphenol (*p* < 0.05). *Thelonectria* was significantly negatively correlated with total carbon, flavone and polysaccharide (*p* < 0.05). *Dactylonectria* was significantly negatively correlated with total carbon flavone and polysaccharide (*p* < 0.01). *Sebacina* was significantly negatively correlated with total carbon, flavone and polysaccharide (*p* < 0.05). *Capnocheirides* was significantly positively correlated with total nitrogen (*p* < 0.05). Fungi_gen_Incertae_sedis was significantly negatively correlated with total phosphorus (*p* < 0.05) (Figure 10a).

*Methyloversatilis* was significantly positively correlated with total nitrogen, total carbon, flavone, and polysaccharide (*p* < 0.01). *Delftia* was significantly positively correlated with total phosphorus, and was significantly negatively correlated with flavone, total carbon and polysaccharide (*p* < 0.01). *Methyloversatilis* was negatively correlated with total phosphorus (*p* < 0.01). *Pseudoalteromonas* and *Vibrio* were significantly positively correlated with total phosphorus (*p* < 0.05). *Kineococcus* was significantly negatively correlated with total nitrogen (*p* < 0.05) (Figure 10b).

### 3.7. PICRUSt and FUNGuild Functional Prediction Analysis

**Figure 11:**
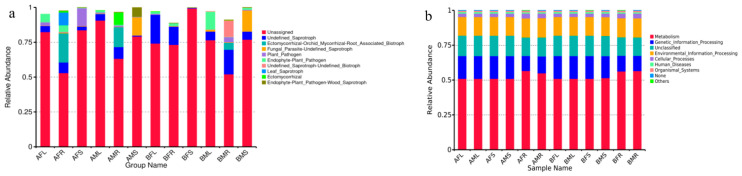
Relative abundance of the predicted trophic mode of fungi (**a**) and relative abundance of predicted KEGG Orthologs functional profiles (KEGG level 1) of bacteria (**b**).

FUNGuild was used to predict the nutritional and functional groups of the fungal communities with different samples. The results showed that nine trophic mode groups could be classified, including Undefined_Saprotroph, Ectomycorrhizal-Orchid_Mycorrhizal-Root_Associated_Biotroph, Fungal_Parasite-Undefined_Saprotroph, Plant_Pathogen, Endophte-Plant_Pathogen, Undefined_Saprotroph-Undefined_Biotroph, Leaf_Saprotroph, Ectomycorrhizal and Endophyte-Plant_Pathogen-Wood_Saprotroph. Ectomycorrhizal was the dominant trophic mode in the AFL and BML samples, accounting for 5.82% and 12.89%, respectively. Ectomycorrhizal-Orchid_Mycorrhizal-Root_Associated_Biotroph was the predominant trophic mode in the AFR and AMR samples, accounting for 20.86% and 14.22%, respectively. Plant_Pathogen was the predominant trophic mode in the AFS sample, accounting for 12.77%, respectively. Undefined_Saprotroph was the predominant trophic mode in the AML, BFL, BFR and BMR samples, accounting for 4.56%, 20.55%, 13.01% and 17.61%, respectively. Fungal_Parasite-Undefined_Saprotroph was the predominant trophic mode in the AMS and BMS samples, accounting for 13.05% and 15.13%, respectively (Figure 11a).

To study the bacterial function, we used PICRUSt to perform bacterial function prediction analysis. Through a comparison with the Kyoto Encyclopedia of Genes and Genomes (KEGG) database, we obtained six primary functional categories of metabolic pathways (primary functional level)—Metabolism, Genetic_Information_Processing, Unclassified, Environment_Information_Processing, Cellular_Processes, Human_Diseases, and Organismal_Systems—and Metabolism was the major component in all samples, accounting for 50.97%~56.62% (Figure 11b).

## 4. Discussion

Dioecious plants constitute highly significant components of terrestrial ecosystems, playing a crucial role in maintaining species diversity and ecosystem stability [45] and also possessing considerable ecological services and economic value for humanity. Owing to long-term adaptive evolution, dioecious plants have exhibited distinct sexual dimorphism in morphology, physiology, and life history [46,47,48,49] and have thereby derived different adaptation mechanisms to environmental changes. Currently, the adaptation mechanisms of dioecious plants to the environment mainly focus on environmental factors, physiology, molecular biology, and chemical defense [50,51,52]. Although these research outcomes have established a certain theoretical and practical basis for uncovering the adaptation mechanism of dioecious plants to the environment, a unified comprehension has not yet been reached, mainly due to the fact that the research focus is often relatively simplistic, and there is a deficiency in comprehensive and systematic exploration of the influence of multiple factors on the adaptation of dioecious plants to the environment. Particularly, the study of the disparities of dioecy-related microorganisms will assist us in revealing the scientific connotation of the adaptation mechanism of dioecious plants to the environment from a novel perspective. Our previous study revealed that significant disparities existed in the rhizosphere microbial diversity, community composition, and co-occurrence network patterns of male and female *H. tibetana* [53]. Endophytes are closely associated with plant growth and stress resistance. Furthermore, are there any disparities in endophytes found in male and female *H. tibetana*? And, if so, what are the factors driving differences? To address these issues, we conducted an analysis of the endophytes of *H. tibetana.* The results indicated that the Shannon index and the Chao1 index of *H. tibetana* in different habitats exhibit significant variations. Additionally, the bacterial diversity in the roots of male plants was observed to be higher than that in the roots of female plants. This finding aligns with previous studies indicating that the root microbial diversity of male plants tends to be greater than that of female plants [54,55,56]. The differences in endophyte diversity between male and female plants may be related to the differences in plant metabolites, thereby causing differences in environmental adaptability between male and female plants.

The dominant phyla of fungi and bacteria were Ascomycetes and Proteobacteria, respectively. Numerous studies have also revealed that in numerous plants, Ascomycetes and Proteobacteria are respectively the dominant phyla of fungal and bacterial endophytes [56,57]. This dominance can be mainly attributed to their superior ecological adaptability. Proteobacteria possess diverse metabolic capabilities, strong motility and colonization potential, and effective strategies to evade plant immune responses, enabling them to thrive inside plant tissues [58]. Ascomycetes produce abundant small spores for efficient dispersal, secrete cell wall-degrading enzymes to penetrate plant tissues, and exhibit flexible trophic lifestyles and strong stress tolerance, facilitating stable endophytic colonization in various plant hosts [59]. At the genus level, the dominant genera of endophytes and their relative abundances vary, which might be attributed to the differences in plant sex [57]. In addition, Chloroflexi and Fusobacteriota were unique endophytic bacteria phyla between male and female individual plants of dioecious *H. tibetana*, respectively. Phylum Chloroflexi is a deep-branching lineage of the domain Bacteria. Chloroflexi microbes are diverse in morphology, nutrition, metabolic pathways, and play important roles in biogeochemical cycles of multiple elements, including carbon, nitrogen and sulfur [60]. Fusobacteriota primarily function as commensals, maintaining ecological balance in polymicrobial communities without causing overt harm to the host in healthy microbiomes [61]. This indicated that there may be some relationship between unique endophytes and plant sex, but the relationship needs to be further explored. LEfSe analysis revealed that the disparities of endophytes in different habitats could serve as potential biomarkers, and these potential biomarkers varied between male and female dioecious *H. tibetana*. In further investigations, these biomarkers might be used as potential indicators of sex-related microbial differences in dioecious *H. tibetana*. In previous studies, it was discovered that the variations in the rhizosphere microbe at diverse levels among dioecious *H. tibetana* plants in different habitats could act as potential biomarkers, and these potential biomarkers differed between male and female *H. tibetana* [53]. The potential biomarkers may be the cause of the differences in environmental adaptability of dioecious *H. tibetana*. The LEfSe analysis only reflects the differences in species abundance and cannot reveal the causal relationships and interaction mechanisms between microorganisms, the environment, and the host. It requires further verification through functional prediction, co-occurrence networks, and in vitro experiments to support the findings. To study endophytes and dioecious *H. tibetana* interactions, a co-occurrence network of microbial communities was constructed. We found that endophytic fungal and bacterial co-occurrence network connectivity and complexity showed differences between females and males of the dioecious *H. tibetana* at different habitats. The result was similar to the reports of Tamang et al. [54]. However, microbial co-occurrence network analysis has inherent limitations. Correlations derived from relative abundance data do not necessarily indicate real biological interactions or causal relationships, and compositional bias and confounding environmental factors may produce spurious associations. Moreover, the network represents a static snapshot of microbial associations rather than dynamic ecological processes. In addition, network construction relies on subjective correlation thresholds, and keystone taxa identified statistically require further experimental validation of their ecological functions [62].

FUNGuild has been employed for the examination of fungal functions, which corresponds to the distinctive functional classification of fungi [63]. Recently, it has been extensively utilized in the analysis of fungal populations. This study discovered that the dominant nutritional patterns of endophytic fungi of dioecious *H. tibetana* in different habitats varied between males and females, suggesting that the nutritional patterns of endophytic fungi were influenced by environmental factors and sex. Endophytic fungi are highly dependent on carbon sources provided by plants (such as photosynthetic products), and at the same time, supply nutrients such as nitrogen and phosphorus to plants [63]. However, the sex differentiation of dioecious plants will significantly change their own resource allocation strategies, thereby altering the nutritional patterns of endophytic fungi. PICRUSt analysis is capable of accurately predicting the metabolic activities of bacterial communities [64]. In this study, we employed high-throughput sequencing data to predict the endophytic bacteria detected by PICRUSt. The results indicate that metabolism constitutes the most significant function in dioecious *H. tibetana*; the reason for this result might be due to the limitations of the PICRUSt database. PICRUSt and FUNGuild are commonly used tools for microbial function prediction, but they have obvious limitations. Both rely on reference databases for indirect prediction rather than directly determining in situ functions. PICRUSt infers gene abundance based on 16S rRNA genes and cannot reflect the actual expression level of genes. Moreover, it has insufficient prediction accuracy for uncultured rare bacteria [43]. FUNGuild has a relatively broad annotation, and it is difficult to precisely classify the actual nutritional types of fungi. A large number of unknown fungi cannot be classified. Therefore, the prediction results only reflect the potential functions of the community and need to be further supported by combining with metagenomics or functional validation experiments [43].

In order to clarify the impact of environmental factors or phytostoichiometry and metabolites on differences of endophytes between females and males of the dioecious *H. tibetana*, VPA studies revealed that phytostoichiometry and metabolites of *H. tibetana* explained more differences in community composition of fungal and bacterial endophytes than rhizosphere soil physicochemical properties. To elucidate the effects of phytostoichiometry and metabolites on the endophyte of *H. tibetana*, Spearman correlation analysis showed that endophytes exhibited a significant positive correlation with the phytostoichiometry and metabolites of *H. tibetana*. Some studies have indicated that the diversity of endophytic bacteria in *Ginkgo biloba* is more accounted for by phytochemical properties rather than environmental factors, and the diversity of the endophytic bacteria community is associated with flavonoids [65]. These results indicated that the differences in endophytes of dioecious *H. tibetana* were influenced by their own phytostoichiometry and metabolites. Therefore, we hypothesize that during the evolution of dioecious plants, there are sex differences in their own metabolites, which lead to differences in endophytes between male and female plants and ultimately result in differences in their adaptability to the environment.

Endophytes perform various important biological functions in plant growth and development. Hence, studying the variations of endophytic communities of male and female plants at reproductive stages helps us understand allocation and adaptation strategies [66]. Our study was limited to clarifying the differences in endophyte diversity and community composition among male and female *H. tibetana*. The driving effect of host gender-specific physiological traits (such as quantitative plant hormones, fingerprinting of root secretions, and monosaccharide profiles of cell walls) on the microbiome, and the effects of endophyte on the growth, development and stress resistance of male and female *H. tibetana*, which need to be explored in the next phase of research.

## 5. Conclusions

In conclusion, this study provided new evidence that there are differences in endophytic microbial communities between females and males of the dioecious *H. tibetana* in different habitats, and unique phyla of endophytes and potential biomarkers of endophytes occur at different levels between females and males of the dioecious *H. tibetana*. Phytostoichiometry and metabolites of *H. tibetana* explained more of the differences in community composition of fungal and bacterial endophytes than those contributed by rhizosphere soil physicochemical properties. Moreover, endophytes exhibited a significant positive correlation with the phytostoichiometry and metabolites of *H. tibetana*. These results highlight the sexual discrimination of endophytes in some dioecious plants and provide important new insights about dioecious plant–microbe interactions.

## Figures and Tables

**Table 1:** The physicochemical characteristics of rhizosphere soils in two habitats.

Sample	TN	TP	TK	QP	QN	QK	OM	SW	SC	pH
AFX	4.61 ± 0.10 b	670.16 ± 8.20 c	17.62 ± 0.17 a	2.71 ± 0.50 b	249.08 ± 21.14 b	288.42 ± 7.82 c	83.22 ± 2.97 b	0.71 ± 0.01 a	0.039 ± 0.00 b	6.86 ± 0.02 a
AMX	4.52 ± 0.00 b	701.46 ± 5.84 b	15.05 ± 0.17 b	3.11 ± 0.13 b	250.99 ± 4.06 b	256.91 ± 4.66 d	84.19 ± 1.41 b	0.71 ± 0.01 a	0.04 ± 0.01 b	6.87 ± 0.01 a
BFX	10.23 ± 0.11 a	848.28 ± 7.07 a	13.83 ± 0.23 c	5.07 ± 0.60 a	606.81 ± 22.80 a	505.66 ± 11.68 a	208.38 ± 0.55 a	0.58 ± 0.12 a	0.068 ± 0.00 a	6.77 ± 0.30 b
BMX	10.03 ± 0.84 a	860.67 ± 3.16 a	11.72 ± 0.64 d	5.10 ± 0.13 a	618.05 ± 8.20 a	472.48 ± 3.50 b	207.93 ± 0.84 a	0.58 ± 0.12 a	0.069 ± 0.00 a	6.76 ± 0.23 b

Note: TN represents total nitrogen, TP represents total phosphorus, TK represents total potassium, QP represents available phosphorus, QN represents available nitrogen, QK represents available potassium, OM represents organic matter, SW represents water content, and SC represents salt content of soil. Different letters above the bars indicate the differences are significant at *p* < 0.05.

**Table 2:** The unique endophytic phyla.

	Sample	Unique Endophytic Phyla		
	AFL	Glomeromycota	Mortierellomycota	
	AML	Rozellomycota	Chytridiomycota	Mucoromycota
	AFS			
	AMS	Mortierellomycota		
	AFR	Rozellomycota		
Fungi	AMR			
	BFL			
	BML			
	BFS	Chytridiomycota		
	BMS			
	BFR			
	BMR	Chytridiomycota		
	AFL			
	AML	Bacteroidota		
	AFS	Acidobacteriota Verrucomicrobiota Bdellovibrionota Patescibacteria	Chloroflexi Abditibacteriota Aenigmarchaeota Gemmatimonadota	Latescibacterota Entotheonellaeota Nitrospirota
	AMS	Desulfobacterota	Fusobacteriota	
	AFR			
Bacteria	AMR	Verrucomicrobiota Acidobacteriota	Myxococcota Patescibacteria Planctomycetota	Desulfobacterota Armatimonadota Dependentiae
	BFL	Bacteroidota	Bdellovibrionota	
	BML			
	BFS	Myxococcota	Bdellovibrionota	Chloroflexi
	BMS	Fusobacteriota Acidobacteriota	Desulfobacterota	Verrucomicrobiota
	BFR	Fibrobacterota	Elusimicrobiota	
	BMR	Deinococcota	Methylomirabilota	

## Data Availability

All raw sequencing data have been submitted to the NCBI Sequence Read Archive (SRA) database under the accession numbers PRJNA1243558 and PRJNA1243560.

## Supplementary Materials

The following supporting information can be downloaded at: https://www.mdpi.com/article/10.3390/microorganisms14061211/s1, Figure S1: Sampling site and sample; Figure S2: The rarefaction curve; Table S1: Topological properties of microbial networks; Table S2: Phytostoichiometry and metabolites of dioecious *H. thibetana* at different habitats.
