# Supplementary material for: Divergence of Dioecious Hippophae tibetana Endophytic Communities and Investigation of Their Key Driving Factors
Source: Microorganisms. 2026 May 27;14(6):1211. doi: 10.3390/microorganisms14061211 (PMC13303047; doi:10.3390/microorganisms14061211)
Supplement: Supplementary file 1 [file microorganisms-14-01211-s001.zip › microorganisms-4318366-supplementary.pdf]

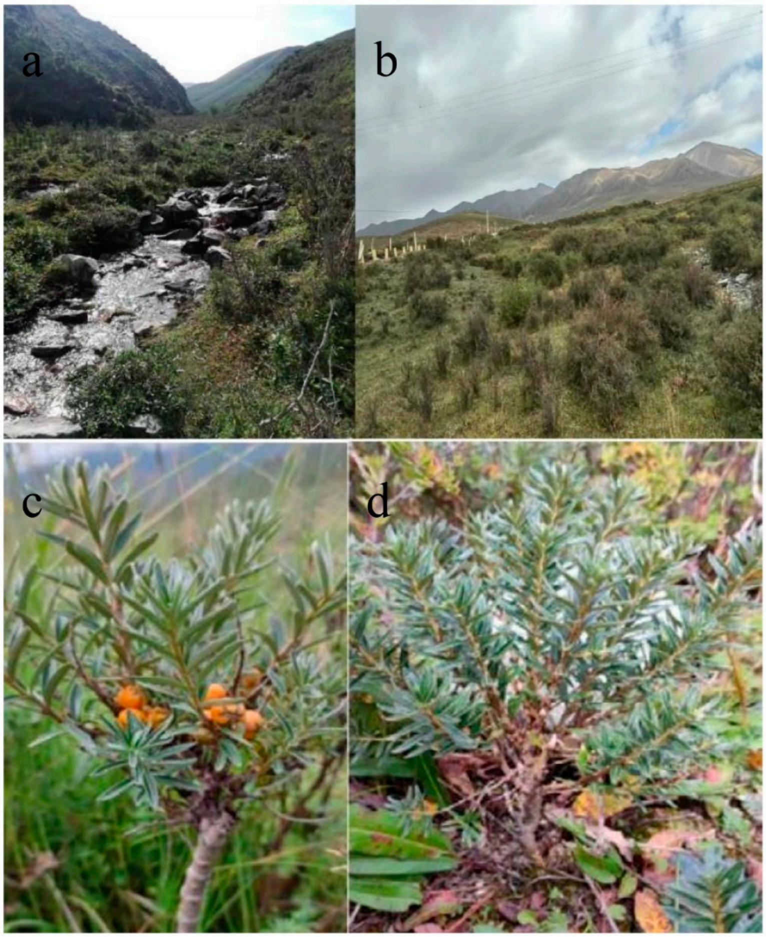

Figure S1. Sampling site and sample

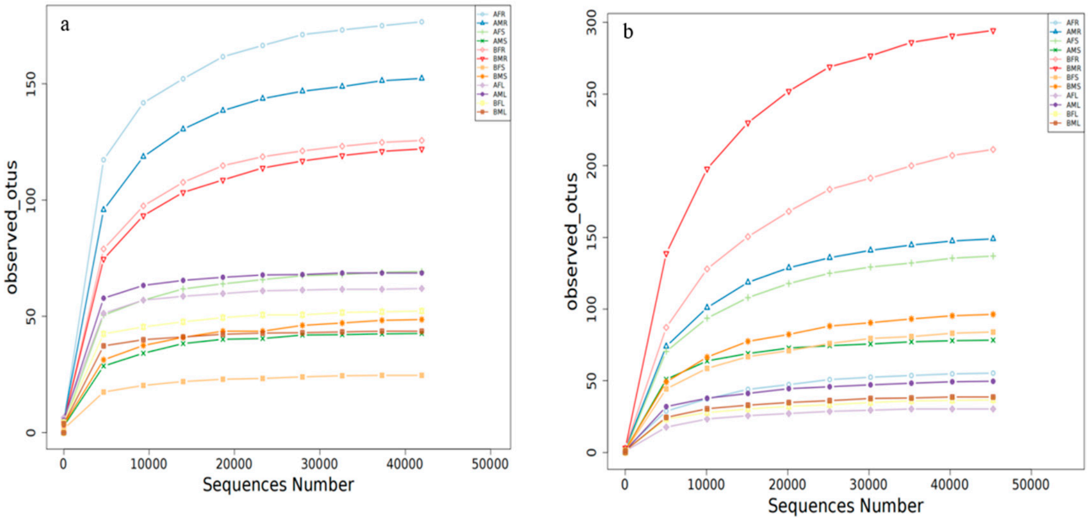

Figure S2. The rarefaction curve

Table S1 Topological properties of microbial networks.

	Sample	Total number of nodes	Total number of links	Positive edges	Negative edges	Relative modularity	Map density
Fungi	AFR	95	1559	1058	285	0.50	0.35
	AMR	96	1943	1657	500	0.59	0.44
	AFS	39	241	157	83	0.63	0.33
	AMS	19	55	42	13	0.58	0.32
	AFL	14	27	15	11	0.56	0.30
	AML	17	45	26	19	0.60	0.33
	BFR	64	716	451	264	0.60	0.36
	BMR	52	455	296	159	0.58	0.34
	BFS	12	21	12	10	0.45	0.32
	BMS	25	101	54	47	0.57	0.34
	BFL	9	16	10	6	0.47	0.29
	BML	18	45	27	18	0.67	0.44
Bacteria	AFR	22	80	46	34	0.48	0.33
	AMR	47	357	223	134	0.62	0.35
	AFS	61	592	321	271	0.67	0.45
	AMS	38	316	197	119	0.23	0.32
	AFL	19	55	29	23	0.32	0.28
	AML	33	145	75	70	0.61	0.32
	BFR	67	759	422	337	0.46	0.34
	BMR	69	852	488	363	0.58	0.36
	BFS	37	260	186	74	0.42	0.36
	BMS	52	481	267	214	0.52	0.39
	BFL	23	79	64	8	0.60	0.31
	BML	28	122	71	58	0.63	0.32

Table S2 Phytostoichiometry and metabolites of dioecious *H. thibetana* at different habitats.

	Total nitrogen (g/kg)	Total phosphorus (g/kg)	Total carbon (%)	Flavone (ug/mg)	Polyphenol (ug/mg)	Polysaccharide (mg/mg)
AML	37.89±0.34g	2.63±0.15b	437.64±0.15d	14.63±0.06h	7.52±0.029h	14.75±0.07i
AFL	35.52±0.95f	1.76±0.14a	433.33±0.14c	13.05±0.06g	7.35±0.049g	18.33±0.04j
BML	30.49±0.55e	1.43±0.04a	450.19±0.07e	11.79±0.008f	4.99±0.029f	15.63±0.08b
BFL	34.67±0.39f	1.37±0.03a	439.25±0.03d	15.56±0.08i	4.81±0.052e	13.88±0.05ab
AMR	24.45±0.45a	4.40±0.005fg	37.56±0.005a	3.85±0.021a	3.38±0.025a	2.32±0.01a
AFR	25.88±0.18cd	4.61±0.033g	41.18±0.03ab	4.06±0.041b	3.46±0.025a	3.04±0.08c
BMR	26.67±0.25d	4.25±0.02ef	40.21±0.02ab	3.86±0.008a	3.43±0.006a	2.58±0.008e
BFR	27.05±0.18d	3.75±0.01c	39.12±0.01ab	4.37±0.023c	3.55±0.030b	2.39±0.023fg
AMS	25.03±0.14cd	3.77±0.03c	41.71±0.03ab	4.56±0.05d	4.24±0.016d	4.91±0.08h
AFS	26.92±0.10d	4.52±0.05g	41.71±0.05ab	4.79±0.02e	3.37.8±0.02a	4.25±0.07f
BMS	24.86±0.10bc	4.15±0.05de	42.34±0.05b	4.85±0.04e	3.83±0.017c	4.45±0.05g
BFS	23.97±0.80ab	3.98±0.04d	42.76±0.04b	4.46±0.02cd	3.36±0.243a	3.29±0.11d
